# Treatment effects in contemporary cardiovascular pharmacotherapy: determinants and interpretation

**DOI:** 10.1093/ehjcvp/pvag043

**Published:** 2026-07-14

**Authors:** Bianca Rocca

**Affiliations:** Department of Medicine and Surgery, LUM University, 70010 Casamassima (Ba), Italy


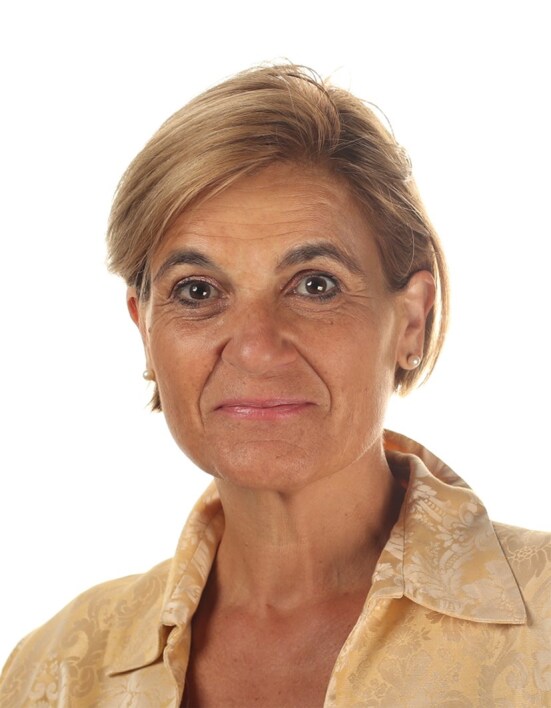


The contributions in this issue of the *European Heart Journal—Cardiovascular Pharmacotherapy* challenge dichotomous interpretations of pharmacotherapy strategies, framed as either intensification or de-escalation, highlighting also the importance of drug tolerability, interactions, persistence, and safety in contemporary populations, while pointing toward emerging therapeutic approaches.

Patients with an acute myocardial infarction (MI) and concomitant atrial fibrillation (AF) face a complex balance between protection from major thromboses and physiological haemostasis. Friberg *et al*.^1^ studied 71 513 survivors of MI with AF identified in Swedish health registries between 2000 and 2021. Temporal patterns in antithrombotic therapy were investigated using 2-year cohorts alongside associated 1-year thrombotic and bleeding outcomes. Notably, treatment patterns evolved markedly over time: while in the earliest cohort (2000–2001) single antiplatelet therapy predominated, in the most recent (2020–2021) cohort direct oral anticoagulant (DOAC)-based strategies (either alone or in combination) prevailed. One-year ischaemic stroke and systemic embolism (SSE) dropped from 6% in the first cohort to 2.1% in the last cohort; cardiovascular mortality was 19.8% and 10.5%, respectively. While temporal associations cannot establish causality, a marked inflexion overlapped with DOAC approval, suggesting a relevant contribution of DOACs to the improved outcomes. However, major bleeding rose over time from 3.9% to a maximum of nearly 8% in the 2014–2015 and 2016–2017 cohorts, declining modestly in subsequent cohorts (6.4% in 2020–2021). A time-matched, non-AF, MI cohort included in the analysis highlights that absolute risks remain higher in AF vs. non-AF patients post-MI, and bleeding remains challenging in this population.

Håkansson *et al*.^2^ investigated ACS patients from the SWEDEHEART registry undergoing drug-coated balloon angioplasty without stent implantation between 2013 and 2022, categorized by intended abbreviated (≤6 months followed by ticagrelor monotherapy) or standard (≥12 months) dual antiplatelet therapy (DAPT) duration. Although not statistically significant, abbreviated DAPT was associated with a consistent trend toward increased all-cause death, stroke and MI at 1 year based on both crude and weighted analyses, with confidence intervals that did not exclude clinically meaningful harm. In-stent restenosis was nearly and significantly 3-fold higher. Bleeding was predominantly Bleeding Academic Research Consortium (BARC) 2 type, limiting the analysis for major bleeding. Thus, the inability to preclude potential harm with abbreviated DAPT challenges the assumptions that abbreviated strategies are generally safer and equally effective. These findings, while hypothesis-generating, call for adequately powered, superiority randomized trials assessing efficacy and safety on hard endpoints.

Clinically relevant drug interactions and treatment persistence contribute to effective cardiovascular pharmacotherapy. Some antiplatelet drugs can be ‘victims’ of pharmacokinetic interactions (e.g. clopidogrel and ticagrelor), while pharmacodynamic interactions occur at the platelet level. Chowdhury *et al*.^3^ investigated 29 973 patients receiving 12-month DAPT following percutaneous coronary intervention (PCI), 3837 (∼13%) with prescriptions of selective serotonin reuptake inhibitors (SSRI; sertraline, paroxetine, fluvoxamine, fluoxetine, citalopram, or escitalopram) before and after PCI. SSRI use was associated with a high risk of major bleeding, mostly intracranial and gastrointestinal, as compared to matched patients without SSRI prescriptions. This may reflect pharmacodynamic interactions, whereby serotonin depletion may impair platelet function, amplifying bleeding diathesis on a background of strong platelet inhibition, as in DAPT, and relevant comorbidities (aging, renal disease, hypertension) or other co-medications affecting platelets (NSAIDs). Pending conclusive evidence, clinicians should implement well-established, guideline-recommended bleeding prevention strategies, including pharmacological gastrointestinal protection and blood pressure optimization, while carefully considering potential pharmacological interactions.

Wu and colleagues addressed a contemporary, relevant polypharmacy issue: the effectiveness and safety of initiating a Sodium-Glucose coTransporter (SGLT)2 inhibitor in 5000 sacubitril/valsartan-treated older adults with heart failure (HF), regardless of diabetes, in a large administrative dataset from Canada from 2019 to 2024. As compared to matched sacubitril/valsartan-only, SGLT2 inhibitor initiation was associated with lower HF hospitalization or all-cause mortality; no major or unexpected safety signals arose from the combination, aside from increased genital infections. Further studies are needed to validate the benefit/risk profile of the combination.

The importance of analytical approaches in interpreting the efficacy in clinical trials is highlighted by Morten Fagerland in his editorial examining the pros and cons of the win ratio methodology.^4^ This approach explicitly imposes a hierarchical structure on composite endpoints. Applied to the Action in Diabetes and Vascular Disease: Preterax and Diamicron Modified Release Controlled Evaluation (ADVANCE) trial by Bompoint *et al*.,^5^ the win ratio approach indicates that cardiovascular death and nephropathy account for the majority of the observed treatment efficacy in the trial, while other components of the primary outcome contributed marginally. However, this apparent clarity in data interpretation introduces a predefined hierarchy, which may itself be a limitation, as summarized in the editorial. The win ratio analysis cannot fully resolve the intrinsic limits of composite endpoints, and a clear interpretation and perspective on the use of this tool as part of clinical trial methodology is provided.

Finally, perspectives on future therapeutics are provided by Giordani *et al*.^6^ and by Lineham *et al*.^7^ Targeted biologic therapies acting on relevant cytokine pathways may suppress myocardial inflammation and spare high-dose steroid use in eosinophilic myocarditis, potentially improving safety.^6^ The focus on venous pathophysiology in HFpEF summarizes potential novel venous therapeutic targets for HF with preserved ejection fraction.^7^

## Data Availability

No data were generated by this work.
